# Prognostic Impact of Early Metabolic Response on Interim 18F-FDG PET/CT in HR+/HER2− Metastatic Breast Cancer Treated with CDK4/6 Inhibitors

**DOI:** 10.3390/medicina62030488

**Published:** 2026-03-05

**Authors:** Vali Aliyev, Ali Kaan Güren, Murad Guliyev, Zeliha Birsin, Murat Günaltılı, Mehmet Cem Fidan, Emir Çerme, Hamza Abbasov, Selin Cebeci, Selver Işık, Murat Sarı, Onur Erdem Şahin, Muhammet Sait Sağer, Özkan Alan, Nebi Serkan Demirci

**Affiliations:** 1 Division of Medical Oncology, Department of Internal Medicine, Cerrahpaşa Faculty of Medicine, Istanbul University-Cerrahpaşa, Istanbul 34098, Türkiye; drguliyev892@gmail.com (M.G.); zelihabirsin@gmail.com (Z.B.); muratgunaltili@hotmail.com (M.G.); mcemfidan@gmail.com (M.C.F.); emircrm34@gmail.com (E.Ç.); hamzaabbasov90@gmail.com (H.A.); sellcebeci@gmail.com (S.C.); ozkan.alan@hotmail.com (Ö.A.); drserkannebi@gmail.com (N.S.D.); 2 Division of Medical Oncology, Department of Internal Medicine, School of Medicine, Marmara University, Istanbul 34899, Türkiye; alikaanguren@gmail.com (A.K.G.); selverr83@gmail.com (S.I.); drmuratsari@gmail.com (M.S.); 3 Department of Nuclear Medicine, Cerrahpaşa Faculty of Medicine, Istanbul University-Cerrahpaşa, Istanbul 34098, Türkiye; onur.sahin@iuc.edu.tr (O.E.Ş.); saitsager@yahoo.com (M.S.S.)

**Keywords:** CDK4/6 inhibitors, ^18F-FDG PET/CT, metabolic response, metastatic breast cancer, SUVmax, prognosis, breast neoplasms

## Abstract

*Background and objectives*: Early biomarkers that can reliably predict treatment outcomes during CDK4/6 inhibitor therapy remain an unmet clinical need in patients with hormone receptor-positive/human epidermal growth factor receptor 2-negative (HR+/HER2−) metastatic breast cancer (MBC). Metabolic changes on ^18F-FDG PET/CT may precede radiologic response and provide insight into tumor biology and early treatment resistance. *Methods*: This two-center retrospective study included 203 patients with HR+/HER2− MBC who received first-line CDK4/6 inhibitors (ribociclib or palbociclib) plus endocrine therapy between 2018 and 2024. Baseline and interim ^18F-FDG PET/CT scans performed after 2–4 cycles were evaluated. Early metabolic response was defined as a ≥30% reduction in SUVmax on the most metabolically active lesion, consistent with PERCIST 1.0. Progression-free survival (PFS) and overall survival (OS) were analyzed using Kaplan–Meier and multivariable Cox models. ROC analysis assessed the discriminative performance of ΔSUVmax for predicting disease progression. *Results*: Among 203 patients, 153 (75.4%) achieved a ≥30% SUVmax reduction. Responders had significantly longer PFS (median 44.4 vs. 4.8 months; *p* < 0.001) and OS (median not reached vs. 32.0 months; *p* < 0.001). Metabolic response remained independently associated with improved PFS (HR 0.24; 95% CI 0.15–0.37; *p* < 0.001) and OS (HR 0.37; 95% CI 0.20–0.67; *p* = 0.001) after adjustment for tumor grade, endocrine resistance, and visceral disease involvement. Non-responders demonstrated more aggressive baseline features, including higher rates of liver (34.0% vs. 15.0%) and brain metastasis (10.0% vs. 1.3%), as well as lower progesterone receptor expression (median 30% vs. 60%). *Conclusions*: Early metabolic response assessed by SUV-max on interim ^18F-FDG PET/CT is independently associated with substantially improved PFS and OS in HR+/HER2− MBC receiving treatment with CDK4/6 inhibitors. Although the predictive accuracy of ΔSUVmax alone was modest, the strong survival gradient suggests meaningful prognostic value. Prospective studies with standardized imaging time points and comprehensive metabolic metrics are warranted to define the role of PET-guided treatment adaptation:

## 1. Introduction

Breast cancer remains the most frequently diagnosed malignancy in women worldwide and continues to account for a substantial proportion of cancer-related deaths [1,2]. Among patients with metastatic disease, the hormone receptor-positive/human epidermal growth factor receptor 2-negative (HR+/HER2−) subtype represents the largest clinical subgroup. Although endocrine therapy (ET) has historically formed the cornerstone of treatment in this setting, resistance—whether present at diagnosis or acquired over time—inevitably limits long-term disease control in many patients [3,4]. The incorporation of cyclin-dependent kinase 4 and 6 (CDK4/6) inhibitors into first-line therapy has significantly extended survival outcomes across multiple randomized phase III trials [5,6,7]. Nevertheless, a subset of patients experiences early progression despite CDK4/6 inhibition [8], underscoring the ongoing need for biomarkers capable of identifying insufficient treatment benefit at an early stage.

Treatment response in metastatic breast cancer is traditionally evaluated using anatomical imaging, where changes in tumor size serve as the primary indicator of therapeutic effect. However, structural regression may occur relatively late, often following earlier biological and metabolic alterations induced by systemic therapy [9]. ^18F-fluorodeoxyglucose positron emission tomography/computed tomography (^18F-FDG PET/CT) offers a quantitative assessment of tumor glucose metabolism and has been increasingly utilized to explore early treatment response in various breast cancer contexts [10,11,12,13,14,15]. The PET Response Criteria in Solid Tumors (PERCIST 1.0) were developed to standardize metabolic response evaluation, shifting the focus from purely morphologic to quantitative metabolic parameters [16].

Several investigations have explored whether baseline metabolic activity or early metabolic changes detected on PET/CT are associated with clinical outcomes in HR+/HER2− metastatic disease. Experimental data further suggest that CDK4/6 inhibition influences cell-cycle regulation and downstream metabolic pathways, potentially altering tumor glucose utilization before measurable tumor shrinkage occurs [17,18]. From a clinical perspective, this raises an important question: can early metabolic shifts observed on PET imaging help distinguish tumors that will derive sustained benefit from CDK4/6 inhibition from those with intrinsic or emerging endocrine resistance?

Most of the existing PET response literature in breast cancer has focused on neoadjuvant or triple-negative settings, where early metabolic suppression has been consistently linked to favorable outcomes [13,14]. In contrast, data addressing metabolic response during CDK4/6 inhibitor therapy in metastatic HR+/HER2− disease remain comparatively sparse. Moreover, previously published studies differ in patient selection, timing of interim imaging, response definitions, and metabolic cut-offs, which limits direct comparability and hinders the establishment of clinically actionable thresholds.

In the present two-center retrospective analysis, we examined whether early metabolic response—defined as a percentage change in SUVmax on interim ^18F-FDG PET/CT performed after 2–4 treatment cycles—was associated with progression-free and overall survival in patients receiving first-line CDK4/6 inhibitor-based therapy for HR+/HER2− metastatic breast cancer. By applying a predefined metabolic threshold aligned with PERCIST principles and evaluating outcomes in a real-world cohort, we sought to clarify the prognostic significance of early PET-based assessment in contemporary clinical practice.

## 2. Materials and Methods

### 2.1. Study Design

This two-center, retrospective cohort study included patients with HR+/HER2− metastatic breast cancer who received first-line endocrine therapy (ET) combined with a CDK4/6 inhibitor (ribociclib or palbociclib) between January 2018 and December 2024. A total of 203 eligible patients were included in the final analysis: 127 from Center 1 and 76 from Center 2. Abemaciclib was not included because it is not reimbursed in our country. Eligible patients were required to have histologically confirmed breast cancer, radiological evidence of metastatic disease, immunohistochemistry (IHC) results for ER, PgR, and HER2, as well as baseline and follow-up ^18F-FDG PET/CT imaging after at least two cycles of CDK4/6 inhibitor therapy. Exclusion criteria included the absence of appropriately timed PET/CT scans, incomplete clinical documentation, lack of measurable metabolic lesions, or insufficient follow-up information.

### 2.2. Patient Population

Clinical and pathological data were extracted from electronic medical records. Baseline characteristics included age, ECOG performance status, menopausal status, tumor histology, metastatic burden, number of metastatic sites, ET backbone, and treatment-related toxicities. Dates of progression, last visit, and death were recorded.

Endocrine sensitivity prior to CDK4/6 inhibitor initiation was defined according to the 5th ESO–ESMO International Consensus Guidelines for Advanced Breast Cancer [19]; recurrence >12 months after adjuvant ET or de novo metastatic presentation was considered endocrine-sensitive, whereas relapse during adjuvant ET or within 12 months of completion was considered endocrine-resistant.

ER, PgR, HER2, and Ki-67 status were obtained from pathology reports of either primary or metastatic samples. For patients with recurrent disease, the biopsy specimen closest to the initiation of CDK4/6 inhibitor therapy was preferentially used. Biomarker evaluation was performed in accordance with ASCO/CAP guidelines [20,21]. HER2-negative disease was defined as IHC 0, and IHC 1+ or 2+ without ISH amplification was categorized as HER2-low.

### 2.3. PET/CT Acquisition and Metabolic Response Assessment

All patients underwent ^18F-FDG PET/CT at baseline and again during early treatment, after 2–4 cycles of CDK4/6 inhibitor therapy (approximately 8–16 weeks). This timing reflected routine clinical practice at the participating centers. Most interim scans were obtained after cycle 3 or 4 (85.7%). Patients fasted for at least 6 h prior to imaging, and serum glucose levels were required to be below 200 mg/dL. A weight-adjusted dose of FDG (3.5–5.0 MBq/kg) was administered intravenously, followed by an uptake period of 50–60 min.

Images were acquired from the skull vertex to the mid-thigh in a craniocaudal direction, with 2–3 min per bed position. Low-dose, non-contrast CT was used for attenuation correction and anatomical correlation.

PET imaging was performed using GE Discovery 710 PET/CT and GE Discovery IQ PET/CT systems (GE Healthcare, Milwaukee, WI, USA) at the two centers. Image reconstruction was carried out using an ordered-subset expectation maximization (OSEM) iterative algorithm (3 iterations, 24 subsets) implemented on the Advantage Workstation software (version 4.7, GE Healthcare, Milwaukee, WI, USA). A 256 × 256 matrix was used, and a 5-mm Gaussian post-reconstruction filter was applied. Although no formal EARL-based harmonization was undertaken in this retrospective setting, the same reconstruction parameters were used at both institutions, which helped limit potential inter-scanner variability.

For response assessment, the most metabolically active lesion on baseline PET/CT was selected and re-evaluated on interim imaging. SUVmax values were recorded for both time points. Metabolic response was defined in line with PERCIST principles, using a ≥30% reduction in SUVmax as the threshold for response [22]. A reduction of <30% or any increase in SUVmax was considered non-response. Importantly, the appearance of new FDG-avid lesions on interim imaging was classified as metabolic progressive disease, regardless of changes observed in the initially selected lesion. Patients with newly detected lesions were therefore categorized as non-responders.

Although PERCIST 1.0 recommends SULpeak for standardized metabolic evaluation, SULpeak measurements were not consistently available across both centers due to differences in software versions and the retrospective nature of data collection. For this reason, SUVmax was used as the primary metabolic parameter. SUVmax is widely applied in retrospective PET analyses and has been shown to correlate closely with SULpeak. The ≥30% reduction threshold was selected to remain consistent with established PERCIST-based response definitions. Nonetheless, we acknowledge that the use of SUVmax instead of SULpeak may introduce some technical variability, which should be considered when interpreting the results.

### 2.4. Efficacy and Safety Measures

Progression-free survival (PFS) was defined as the interval from treatment initiation to radiological or clinical progression or death. Overall survival (OS) was defined as the time from treatment initiation until death or last follow-up. Adverse events were graded using the National Cancer Institute Common Terminology Criteria for Adverse Events (CTCAE) version 5.0 [23].

### 2.5. Ethical Considerations

The study was approved by the institutional ethics committee (Approval No.: E-74555795-050.04-1419635, dated 17 September 2025). Informed consent was waived due to the retrospective study design.

### 2.6. Statistical Analysis

Statistical analyses were performed using SPSS version 27. The Kolmogorov–Smirnov test was used to evaluate the distribution of continuous variables. Normally distributed variables were compared using the t-test; non-normally distributed variables were analyzed using the Mann–Whitney U test. Categorical variables were compared using chi-square or Fisher’s exact test. Survival analyses were performed using the Kaplan–Meier method, and differences were assessed with the log-rank test. Multivariate Cox regression analysis was conducted to identify independent prognostic factors. Variables with *p* < 0.10 in univariate analysis or those considered clinically relevant were included in the multivariable Cox regression model. A two-sided *p*-value < 0.05 was considered statistically significant.

## 3. Results

### 3.1. Baseline Characteristics of the Patients

A total of 203 patients with HR+/HER2− metastatic breast cancer met the inclusion criteria and underwent both baseline and interim ^18F-FDG PET/CT. Of these, 127 were treated at Center 1 and 76 at Center 2. The median age was 56 years (range, 27–87), and the majority of patients were postmenopausal (70.0%) and had an ECOG performance score of 0 (62.1%). Invasive ductal carcinoma was the predominant histologic subtype (71.8%).

Overall, 153 patients (75.4%) achieved a ≥30% reduction in SUVmax, whereas 50 patients (24.6%) did not. Non-responders had significantly lower PgR expression (median 30% vs. 60%, *p* = 0.004) and were more likely to have liver (34.0% vs. 15.0%, *p* = 0.007) and brain metastasis (10.0% vs. 1.3%, *p* = 0.011). Bone-only metastasis was more frequent among responders (30.7% vs. 16.0%, *p* = 0.045). Baseline characteristics are summarized in Table 1.

### 3.2. Survival Outcomes

At the time of data cutoff, the median follow-up duration was 25.2 months (range, 3.1–82.6). Overall, 92 patients (45.3%) experienced disease progression and 49 patients (24.1%) had died. Progression was significantly more frequent in metabolic non-responders compared with responders (80.0% vs. 33.9%, *p* < 0.001). Likewise, mortality was also markedly higher among non-responders than responders (46.0% vs. 17.0%, *p* < 0.001).

#### 3.2.1. Progression-Free Survival

**Figure 1:**
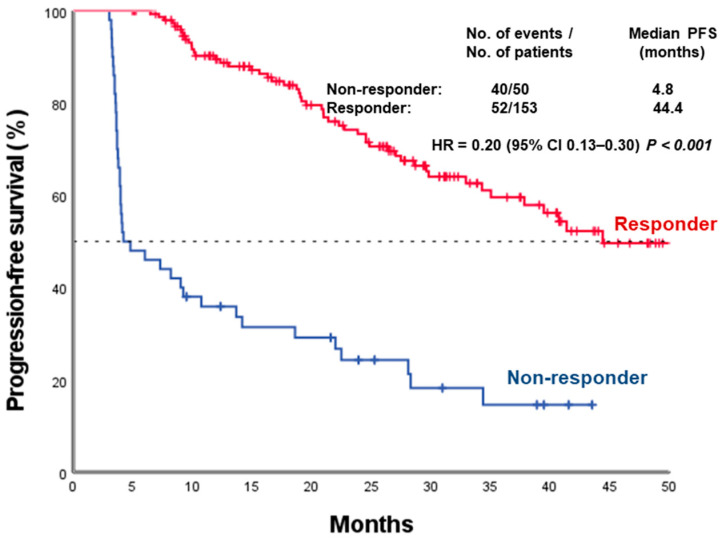
Kaplan–Meier curves for progression-free survival according to metabolic response. PFS: progression-free survival; CI: confidence interval; HR: hazard ratio.

Patients who achieved a metabolic response demonstrated markedly prolonged PFS compared with non-responders (median 44.4 vs. 4.8 months; log-rank *p* < 0.001) (Figure 1). The PFS curves separated early and remained widely divergent throughout the entire follow-up period.

In univariate analysis, high tumor grade, endocrine-resistant disease, recurrent presentation, visceral involvement, liver metastasis, and brain metastasis were all associated with inferior PFS. Variables with *p* < 0.10, as well as those considered clinically relevant, were included in the multivariable model.

**Figure 2:**
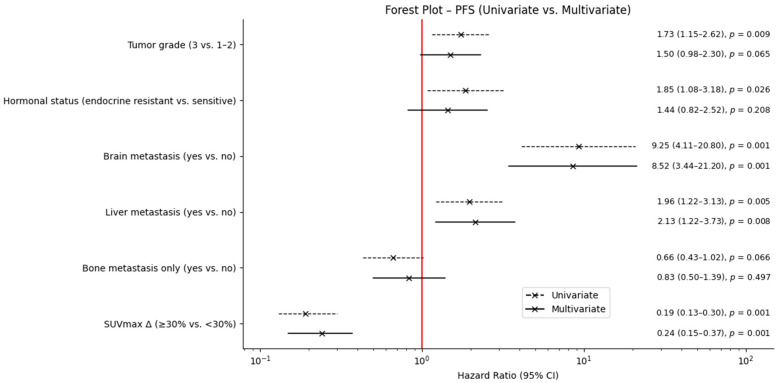
Forest plot demonstrating multivariate Cox regression results for progression-free survival. PFS: progression-free survival; HR: hazard ratio; CI: confidence interval; TRAE: treatment-related adverse event; SUVmax Δ: percentage change in maximum standardized uptake value.

In multivariable Cox regression, metabolic response remained independently associated with improved PFS (HR 0.24; 95% CI 0.15–0.37; *p* < 0.001). Brain metastasis (HR 8.52) and liver metastasis (HR 2.13) emerged as the strongest predictors of shortened PFS. Full results are shown in Table 2 and Figure 2.

#### 3.2.2. Overall Survival

**Figure 3:**
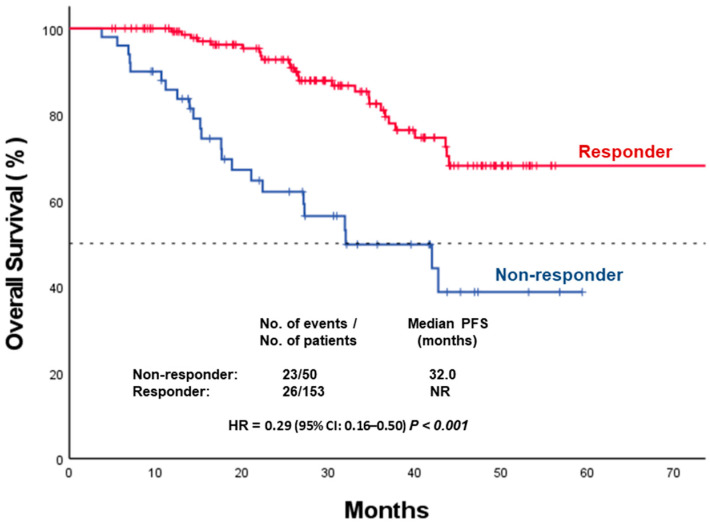
Kaplan–Meier curves for overall survival according to metabolic response. PFS: progression-free survival; CI: confidence interval; HR: hazard ratio.

Metabolic responders had significantly improved OS compared with non-responders (median OS not reached vs. 32.0 months; *p* < 0.001) (Figure 3).

In univariate analysis, high tumor grade (*p* = 0.049), liver metastasis (*p* < 0.001), and brain metastasis (*p* < 0.001) were each associated with worse OS, whereas premenopausal status was not significantly associated with survival (*p* = 0.738).

**Figure 4:**
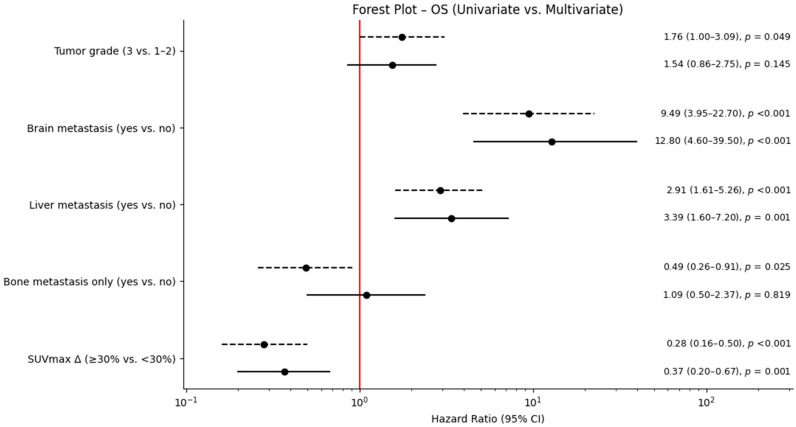
Forest plot demonstrating multivariate Cox regression results for overall survival. OS: overall survival; HR: hazard ratio; CI: confidence interval SUVmax Δ: percentage change in maximum standardized uptake value.

In multivariable analysis, metabolic response remained independently prognostic for OS (HR 0.37; 95% CI 0.20–0.67; *p* = 0.001). Brain metastasis (HR 12.8) and liver metastasis (HR 3.39) were the strongest predictors of mortality. Results are summarized in Table 3 and Figure 4.

Survival outcomes did not significantly differ between centers (log-rank *p* = 0.62; Supplementary Figure S4).

In center-stratified analyses, the association between metabolic response and progression-free survival remained consistent across both institutions. Among patients treated at Center 1, metabolic responders demonstrated significantly improved PFS compared to non-responders (HR 0.47, *p* < 0.001). Similarly, in Center 2, metabolic response was significantly associated with prolonged PFS (HR 0.33, *p* < 0.001) (Supplementary Table S3).

Baseline SUVmax was not significantly associated with PFS or OS, whereas interim SUVmax showed a significant association with both outcomes (Supplementary Table S4).

When stratified by metabolic response category (CR/PR/SD/PD) on interim PET/CT, significant differences in progression-free survival were observed (log-rank *p* < 0.001). CR/PR were associated with the most favorable outcomes, whereas PD showed the poorest prognosis (Supplementary Table S5 and Figure S5).

### 3.3. Treatment Exposure

Of the 203 patients, 149 (73.4%) received ribociclib and 54 (26.6%) received palbociclib. Most patients were treated with an aromatase inhibitor (79.8%), whereas 20.2% received fulvestrant. Baseline characteristics were similar between ribociclib- and palbociclib-treated patients. No significant differences in survival outcomes were observed between the two CDK4/6 inhibitors (PFS *p* = 0.61; OS *p* = 0.49). Detailed treatment distributions and Kaplan–Meier curves comparing ribociclib and palbociclib are provided in Supplementary Table S1 and Supplementary Figure S1. Treatment distribution did not differ meaningfully between the responder and non-responder groups.

### 3.4. Treatment-Related Adverse Events

A total of 80 patients (40.4%) experienced at least one TRAE. Neutropenia was the most frequent event (37.4%), followed by thrombocytopenia (4.4%) and anemia (3.0%). A complete toxicity summary is provided in Table 4.

Dose reduction occurred in 33.8% of patients, primarily due to neutropenia. There was no significant difference in TRAE rates between responders and non-responders.

## 4. Discussion

In this two-center real-world cohort, early metabolic changes observed on interim ^18F-FDG PET/CT were closely associated with long-term outcomes in patients with HR+/HER2− metastatic breast cancer treated with first-line CDK4/6 inhibitors. With a median follow-up of 25.2 months, the dataset allowed for a meaningful assessment of both progression and survival endpoints. A reduction of at least 30% in SUVmax after 2–4 treatment cycles consistently identified patients who experienced substantially longer progression-free and overall survival.

These findings reinforce the concept that metabolic alterations often occur earlier than measurable tumor shrinkage on anatomical imaging, offering a window into treatment sensitivity before conventional radiologic changes become evident [24,25,26]. In our supplementary analysis, the ROC curve demonstrated only moderate discriminatory capacity (AUC 0.65; Supplementary Figure S3). However, ΔSUVmax showed a much clearer separation when evaluated within time-to-event survival models, suggesting that its clinical value lies more in prognostic stratification than in binary response classification.

Despite the survival gains achieved with CDK4/6 inhibitors, early disease progression remains a clinically relevant issue, particularly in tumors characterized by intrinsic endocrine resistance. This underscores the importance of identifying markers capable of signaling insufficient treatment benefit before overt radiologic progression occurs. Metabolic imaging has been proposed as one such approach, given that alterations in tumor glucose utilization may reflect pharmacodynamic effects earlier than measurable size reduction [27,28].

The prospective PUCCINI study, which specifically evaluated PET response during CDK4/6 inhibitor therapy, demonstrated that metabolic suppression after two treatment cycles was associated with improved progression-free survival, independent of traditional prognostic factors [25]. In our cohort, a similar pattern was observed under routine clinical conditions. The consistent association between ΔSUVmax and survival—maintained even after adjusting for visceral disease burden and endocrine resistance—supports the notion that early metabolic change captures biologically meaningful treatment sensitivity rather than merely reflecting baseline risk characteristics.

The existing literature evaluating PET-based response during CDK4/6 inhibitor therapy in HR+/HER2− metastatic breast cancer remains limited and methodologically heterogeneous. Several published analyses differ substantially with regard to treatment setting (first-line vs. later-line), sample size, imaging time points, and metabolic response definitions. In many studies, interim PET was performed at variable intervals, and response thresholds were not consistently aligned with PERCIST criteria. Furthermore, most prior investigations primarily reported progression-free survival, whereas mature overall survival data were either unavailable or insufficiently powered.

These methodological inconsistencies complicate cross-study comparisons and limit the establishment of clinically actionable metabolic cut-offs. In contrast, our analysis focuses exclusively on patients treated in the first-line setting with CDK4/6 inhibitor-based therapy, applies a predefined ≥30% SUVmax reduction threshold consistent with PERCIST principles, and evaluates both progression-free and overall survival in a unified real-world cohort. By integrating these elements, the present study aims to provide a more standardized and clinically interpretable assessment of early metabolic response.

Outside the context of randomized trials, several real-world investigations have examined metabolic changes during CDK4/6 inhibitor therapy. Retrospective reports indicate that patients who achieve metabolic suppression tend to experience longer disease control and improved survival compared with those who do not [18,29]. Similar observations have also been described in endocrine-resistant or heavily pretreated populations, supporting the idea that metabolic response may reflect underlying tumor biology more directly than purely size-based RECIST measurements [30].

In our cohort, patients classified as metabolic non-responders more frequently exhibited features associated with aggressive disease, including liver and brain metastases, as well as lower PgR expression. These characteristics have previously been linked to reduced sensitivity to CDK4/6 inhibition [31,32]. Although the number of patients with brain metastases was limited (n = 7), the direction and magnitude of the observed effect were in line with prior data describing the unfavorable prognosis of this subgroup.

Biological data provide additional context for these clinical observations. Experimental models suggest that CDK4/6 inhibition affects cellular metabolism by reducing glycolytic activity and altering glucose uptake pathways [33]. Moreover, genomic alterations commonly associated with endocrine resistance—such as ESR1 mutations, activation of the PI3K/AKT pathway, and RB1 loss—have been linked to enhanced glucose metabolism and diminished responsiveness to CDK4/6 blockade [34,35]. These mechanistic insights are compatible with our clinical findings, in which limited metabolic suppression on interim PET/CT was associated with more aggressive disease behavior.

Although ribociclib and palbociclib are categorized within the same CDK4/6 inhibitor class, they differ in pharmacologic properties, selectivity patterns, dosing strategies, and toxicity profiles. In our cohort, survival outcomes did not significantly differ between the two agents. Nevertheless, subtle biological differences between these compounds cannot be completely ruled out and may warrant further investigation in larger comparative analyses.

Several aspects of the current analysis deserve consideration. The study reflects real-world clinical practice and includes a relatively large cohort of patients treated uniformly in the first-line metastatic setting. Both baseline and interim PET/CT examinations were available for all included patients, allowing a consistent assessment of metabolic change over time. The combination of structured imaging assessment with detailed clinical and pathological data enabled a comprehensive evaluation of the prognostic implications of early metabolic response.

## 5. Limitations

This analysis should be interpreted in light of several methodological constraints. First, the retrospective design inherently carries the possibility of selection bias and incomplete data capture. Imaging was performed at two institutions using different PET systems, and no formal cross-calibration strategy was implemented. Although reconstruction parameters were identical and metabolic change was evaluated as an intra-patient percentage variation (ΔSUVmax), some degree of measurement variability cannot be entirely excluded. Importantly, center-stratified analyses yielded comparable results, suggesting that scanner-related differences were unlikely to have driven the main survival associations.

Metabolic response was assessed using SUVmax rather than the PERCIST-preferred SULpeak because SULpeak extraction was not consistently feasible in this retrospective multicenter dataset. In addition, interim PET/CT examinations were obtained between the second and fourth treatment cycles, reflecting routine practice rather than a strictly predefined imaging schedule. This temporal variability may have influenced the magnitude of observed metabolic changes. The use of a single target lesion per patient also limits the ability to account for intrapatient metabolic heterogeneity.

Only a small proportion of patients had baseline brain metastases (3.4%), which restricts the strength of conclusions for individuals with central nervous system involvement. Comprehensive molecular profiling—including ESR1, PIK3CA, and RB1 status—was not uniformly available, preventing a detailed exploration of genomic correlates of metabolic response. Furthermore, volumetric PET parameters such as metabolic tumor volume (MTV) and total lesion glycolysis (TLG) were not systematically collected and therefore could not be incorporated into the analysis.

Formal internal validation procedures (such as bootstrapping or cross-validation) and independent external validation were not performed due to the retrospective design and sample size constraints. Therefore, the generalizability and reproducibility of these findings should be interpreted cautiously and require confirmation in prospective external cohorts.

Prospective studies with standardized imaging schedules, harmonized acquisition parameters, and integrated molecular characterization would be valuable to confirm and extend these findings. While these limitations should be considered when interpreting the results, the consistency of survival associations across two independent centers provides reassurance regarding the stability of the observed effect.

## 6. Conclusions

Early metabolic response assessed by interim ^18F-FDG PET/CT is a strong and independent predictor of progression-free and overall survival in patients with HR+/HER2− metastatic breast cancer treated with first-line CDK4/6 inhibitors. A ≥30% reduction in SUVmax reliably identified patients with markedly better outcomes, and the prognostic value of this metabolic response remained consistent even after adjustment for established clinical risk factors. These findings suggest that interim PET/CT may serve as a valuable tool for early treatment evaluation, helping to identify patients at high risk for early disease progression who may require closer monitoring. Prospective studies using standardized PET acquisition protocols and integrated molecular analyses are warranted to confirm these results and to define the optimal role of metabolic response in guiding personalized treatment strategies.

## Figures and Tables

**Table 1:** Baseline demographic and clinical characteristics stratified by metabolic response (SUVmax <30% vs. ≥30%).

Variables		All Patients	<30 SUVmax	≥30 SUVmax	*p*-Value
		(n = 203)	(n = 50)	(n = 153)	
Age (years)	Median (range)	56 (27–87)	57 (32–82)	56(27–87)	0.871
ECOG PS, n (%)	0	126 (62.1)	28 (56.0)	98 (64.1)	0.319
	≥1	77 (37.9)	22 (44.0)	55 (35.9)	
Menopausal status, n (%)	Premenopausal	61 (30.0)	15 (30.0)	46 (30.1)	0.993
	Postmenopausal	142 (70.0)	35 (70.0)	107 (69.9)	
Histopathology, n (%)	IDC	145 (71.8)	37 (74.0)	108 (71.1)	0.149
	ILC	18 (8.9)	7 (14.0)	11 (7.2)	
	Other	39 (19.3)	6 (12.0)	33 (21.7)	
Estrogen receptor level (%)	Median (range)	95 (10–100)	90 (30–100)	95 (10–100)	0.071
Progesterone receptor level (%)	Median (range)	60 (0–100)	30 (0–100)	60 (0–100)	0.004
Ki 67 (%)	Median (range)	25 (3–90)	30 (3–80)	21 (5–90)	0.280
	≥20, n (%)	129 (63.5)	32 (64.0)	97 (63.4)	0.995
Tumor grade	Grade 1–2	97 (63.0)	24 (55.8)	73 (67.0)	0.261
	Grade 3	55 (36.2)	19 (44.2)	36 (33.0)	
HER2 status, n (%)	Zero	142 (70.0)	32 (64.0)	110 (71.9)	0.293
	Low	61 (30.0)	18 (36.0)	43 (28.1)	
Metastatic disease, n (%)	De novo	109 (53.7)	18 (36.0)	91 (59.5)	0.005
	Recurrent	94 (46.3)	32 (64.0)	62 (40.5)	
Hormonal status, n (%)	Endocrine sensitive	175 (86.2)	39 (78.0)	136 (88.9)	0.061
	Endocrine resistance	28 (13.8)	11 (22.0)	17 (11.1)	
CDK4/6 inhibitors, n (%)	Ribociclib	149 (73.4)	38 (76.0)	111 (72.5)	0.714
	Palbociclib	54 (26.6)	12 (24.0)	42 (27.5)	
Endocrine therapy, n (%)	Aromatase inhibitor	162 (79.8)	35 (70.0)	127 (83.0)	0.066
	Fulvestrant	41 (20.2)	15 (30.0)	26 (17.0)	
Bone lesion only, n (%)		55 (27.1)	8 (16.0)	47 (30.7)	0.045
Lymph node metastasis, n (%)		83 (40.9)	21 (42.0)	62 (40.5)	0.870
Visceral metastasis, n (%)		93 (45.8)	30 (60.0)	63 (41.2)	0.023
Lung metastasis, n (%)		55 (27.1)	12 (24.0)	43 (28.1)	0.714
Liver metastasis, n (%)		40 (19.7)	17 (34.0)	23 (15.0)	0.007
Brain metastasis, n (%)		7 (3.4)	5 (10.0)	2 (1.3)	0.011
Any grade TRAE, n (%)		80 (40.4)	17 (34.7)	63 (42.3)	0.403
Dose-reducing TRAE, n (%)		68 (33.8)	18 (36.7)	50 (32.9)	0.729

IDC: invasive ductal carcinoma; ILC: invasive lobular carcinoma; ER: estrogen receptor; HER2: human epidermal growth factor receptor 2; ECOG PS: Eastern Cooperative Oncology Group Performance Score; AI: aromatase inhibitor; ET: endocrine therapy; SUVmax: maximum standardized uptake value.

**Table 2:** Univariate and multivariate Cox regression analyses for progression-free survival.

	Univariate			Multivariate		
Variable	HR	95% CI	*p*-Value	HR	95% CI	*p*-Value
Age (≥65 vs. <65 years)	0.77	0.48–1.23	0.269	-	-	-
Menopausal status (post vs. pre)	0.87	0.56–1.36	0.552	-	-	-
Ki-67 (≥20 vs. <20%)	1.16	0.75–1.80	0.507	-	-	-
Tumor grade (3 vs. 1–2)	1.73	1.15–2.62	0.009	1.50	0.98–2.30	0.065
HER2 status (low vs. zero)	1.09	0.69–1.73	0.718	-	-	-
Metastatic disease (recurrent vs. de novo)	1.39	0.92–2.09	0.120	-	-	-
Hormonal status (endocrine resistant vs. sensitive)	1.85	1.08–3.18	0.026	1.44	0.82–2.52	0.208
CDK 4/6 inhibitors (palbociclib vs. ribociclib)	0.89	0.56–1.41	0.608	-	-	-
Brain metastasis (yes vs.no)	9.25	4.11–20.8	<0.001	8.52	3.44–21.2	<0.001
Liver metastasis (yes vs. no)	1.96	1.22–3.13	0.005	2.13	1.22–3.73	0.008
Lung metastasis (yes vs. no)	0.82	0.51–1.32	0.407	-	-	-
Bone metastasis only (yes vs. no)	0.66	0.43–1.02	0.066	0.83	0.50–1.39	0.497
Any-grade TRAE (yes vs. no)	1.09	0.72–1.65	0.668	-	-	-
Dose-reducing TRAE (yes vs. no)	1.18	0.78–1.79	0.421	-	-	-
SUVmax Δ (≥30% vs. <30%)	0.19	0.13–0.30	<0.001	0.24	0.15–0.37	<0.001

HR: hazard ratio; CI: confidence interval; PFS: progression-free survival; TRAE: treatment-related adverse event; HER2: human epidermal growth factor receptor 2; ET: endocrine therapy; SUVmax Δ: percentage change in maximum standardized uptake value.

**Table 3:** Univariate and Multivariate Cox Regression Analysis for Overall Survival.

	Univariate			Multivariate		
Variable	HR	95% CI	*p*-Value	HR	95% CI	*p*-Value
Age (≥65 vs. <65 years)	1.14	0.62–2.10	0.665	-	-	-
Menopausal status (pre vs. post)	1.11	0.59–2.10	0.738	-	-	-
Ki-67 (≥20 vs. <20%)	0.86	0.48–1.53	0.613	-	-	-
Tumor grade (3 vs. 1–2)	1.76	1.00–3.09	0.049	1.54	0.86–2.75	0.145
HER2 status (low vs. zero)	0.84	0.53–1.31	0.451	-	-	-
Metastatic disease (recurrent vs. de novo)	1.29	0.71–2.18	0.436	-	-	-
Hormonal status (endocrine resistant vs. sensitive)	1.69	0.81–3.49	0.156	-	-	-
CDK 4/6 inhibitors (palbociclib vs. ribociclib)	0.79	0.42–1.51	0.490	-	-	-
Brain metastasis (yes vs.no)	9.49	3.95–22.7	<0.001	12.8	4.60–39.5	<0.001
Liver metastasis (yes vs. no)	2.91	1.61–5.26	<0.001	3.39	1.60–7.20	0.001
Lung metastasis (yes vs. no)	0.69	0.34–1.38	0.296	-	-	-
Bone metastasis only (yes vs. no)	0.49	0.26–0.91	0.025	1.09	0.50–2.37	0.819
Any-grade TRAE (yes vs. no)	1.02	0.57–1.84	0.923	-	-	-
Dose-reducing TRAE (yes vs. no)	1.20	0.68–2.11	0.514	-	-	-
SUVmax Δ (≥30% vs. <30%)	0.28	0.16–0.50	<0.001	0.37	0.20–0.67	0.001

HR: hazard ratio; CI: confidence interval; OS: overall survival; TRAE: treatment-related adverse event; HER2: human epidermal growth factor receptor 2; ET: endocrine therapy; SUVmax Δ: percentage change in maximum standardized uptake value.

**Table 4:** Treatment-related adverse events (any grade and dose-reducing) during CDK4/6 inhibitor therapy.

Adverse Event	Any Grade, n (%)	Dose-Reducing n (%)
Neutropenia	76 (37.4%)	63 (31.0%)
Thrombocytopenia	9 (4.4%)	7 (3.4%)
Anemia	6 (3.0%)	4 (2.0%)
QTc prolongation	7 (3.4%)	7 (3.4%)
ALT/AST increased	3 (1.5%)	3 (1.5%)
Creatinine increased	1 (0.5%)	1 (0.5%)
Diarrhea	2 (1.0%)	2 (1.0%)
Oral mucositis	1 (0.5%)	1 (0.5%)

ALT: alanine aminotransferase; AST: aspartate aminotransferase; QTc: corrected QT interval.

## Data Availability

The datasets analyzed during the current study are available from the corresponding author upon reasonable request.

## Supplementary Materials

The following supporting information can be downloaded at: https://www.mdpi.com/article/10.3390/medicina62030488/s1, Table S1: Baseline clinical and demographic characteristics according to CDK4/6 inhibitor type. Table S2: Metabolic response rates across metastatic subgroups. Table S3: Center-stratified analysis of the association between metabolic response and progression-free survival. Table S4: Univariable Cox regression analysis evaluating baseline and interim SUVmax in relation to progression-free survival (PFS) and overall survival (OS). Table S5: Distribution of metabolic response categories on interim PET/CT and associated progression-free survival. Figure S1: Kaplan–Meier curve for progression-free survival according to CDK4/6 inhibitor type. Figure S2: Histogram showing the distribution of percentage change in SUVmax among responders and non-responders. Figure S3: Receiver operating characteristic (ROC) curve demonstrating the discriminative performance of ΔSUVmax for predicting disease progression. Figure S4: Kaplan–Meier curves for progression-free survival according to study center. Figure S5: Kaplan–Meier curves for progression-free survival according to metabolic response category (CR, PR, SD, PD) on interim PET/CT.
