# Supplementary material for: Prognostic Impact of Early Metabolic Response on Interim 18F-FDG PET/CT in HR+/HER2− Metastatic Breast Cancer Treated with CDK4/6 Inhibitors
Source: Medicina (Kaunas). 2026 Mar 5;62(3):488. doi: 10.3390/medicina62030488 (PMC13027895; doi:10.3390/medicina62030488)
Supplement: Supplementary file 1 [file medicina-62-00488-s001.zip › medicina-4118162-supplementary.pdf]

Supplementary Table S1. Baseline characteristics by CDK4/6 inhibitor type

Variable	All Patients (n=203)	Ribociclib (n=149)	Palbociclib (n=54)	p-Value
**Age (years), median (range)**	56 (27–87)	55 (27–82)	58 (34–87)	**0.014**
**ECOG performance status, n (%)**				0.071
0	126 (62.1)	99 (66.4)	27 (50.0)	
≥1	71 (35.0)	47 (31.5)	24 (44.4)	
**Menopausal status, n (%)**				0.102
Pre	61 (30.0)	50 (33.6)	11 (20.4)	
Post	142 (70.0)	99 (66.4)	43 (79.6)	
**Histopathology, n (%)**				0.078
IDC	145 (71.4)	108 (72.5)	37 (68.5)	
ILC	18 (8.9)	16 (10.7)	2 (3.7)	
Other	39 (19.7)	24 (16.1)	15 (27.8)	
**Estrogen receptor (%), median (range)**	95 (10–100)	95 (10–100)	95 (50–100)	0.207
**Progesterone receptor (%), median (range)**	60 (0–100)	50 (0–100)	70 (0–100)	0.144
**Ki-67 (%), median (range)**	25 (3–90)	25 (3–90)	20 (5–70)	0.165
**Ki-67 ≥20%, n (%)**	129 (63.5)	101 (67.8)	28 (51.9)	0.163
**Tumor grade (1–2), n (%)**	97 (63.8)	68 (60.2)	29 (74.4)	0.163
**Endocrine-resistant disease, n (%)**	28 (13.8)	25 (16.8)	3 (5.6)	0.069

Variable	All Patients (n=203)	Ribociclib (n=149)	Palbociclib (n=54)	p-Value
**De novo metastatic disease, n (%)**	109 (53.7)	78 (52.3)	31 (57.4)	0.632
**HER2 status, n (%)**				0.394
Zero	142 (70.0)	102 (68.5)	40 (74.1)	
Low	61 (30.0)	47 (31.5)	14 (25.9)	
**Bone-only metastasis, n (%)**	55 (27.1)	41 (27.5)	14 (25.9)	0.963
**Visceral metastasis, n (%)**	99 (48.8)	69 (46.3)	30 (55.6)	0.315

Comment: Baseline characteristics were generally balanced between treatment groups. Patients receiving palbociclib tended to be slightly older and more frequently postmenopausal, but other clinicopathological parameters were broadly comparable.

Supplementary Figure S1. Kaplan–Meier curve for progression-free survival according to CDK4/6 inhibitor type

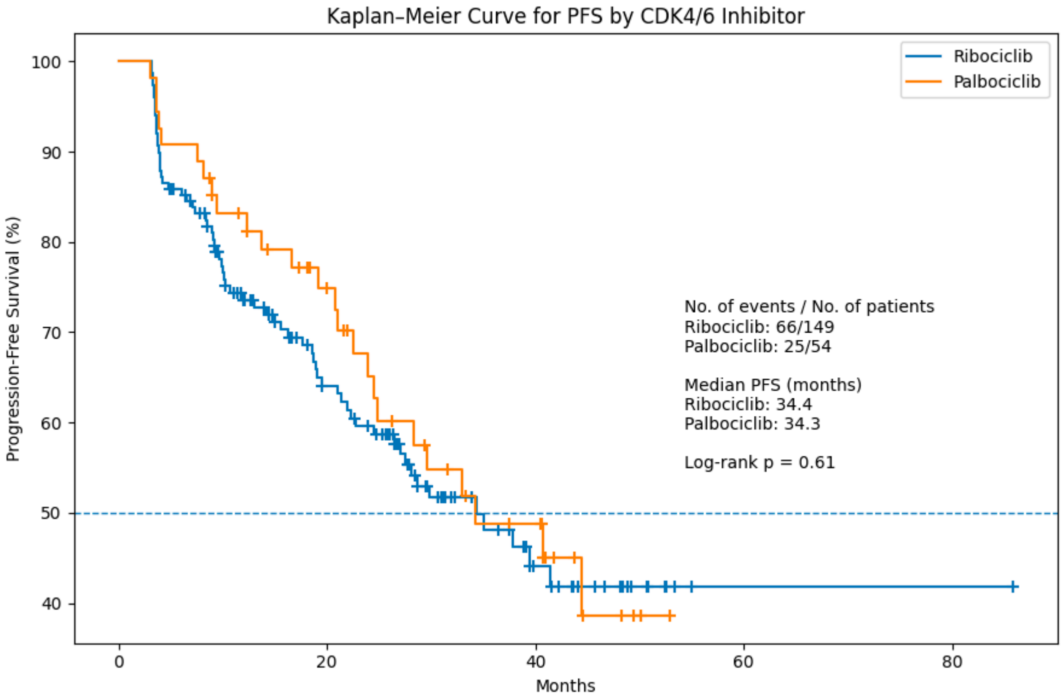

No significant difference in PFS was observed between patients treated with ribociclib and palbociclib (log-rank p = 0.61). Median PFS was 34.4 months for ribociclib and 34.3 months for palbociclib. Tick marks indicate censored observations.

Supplementary Figure S2. Histogram of Percentage Change in SUVmax

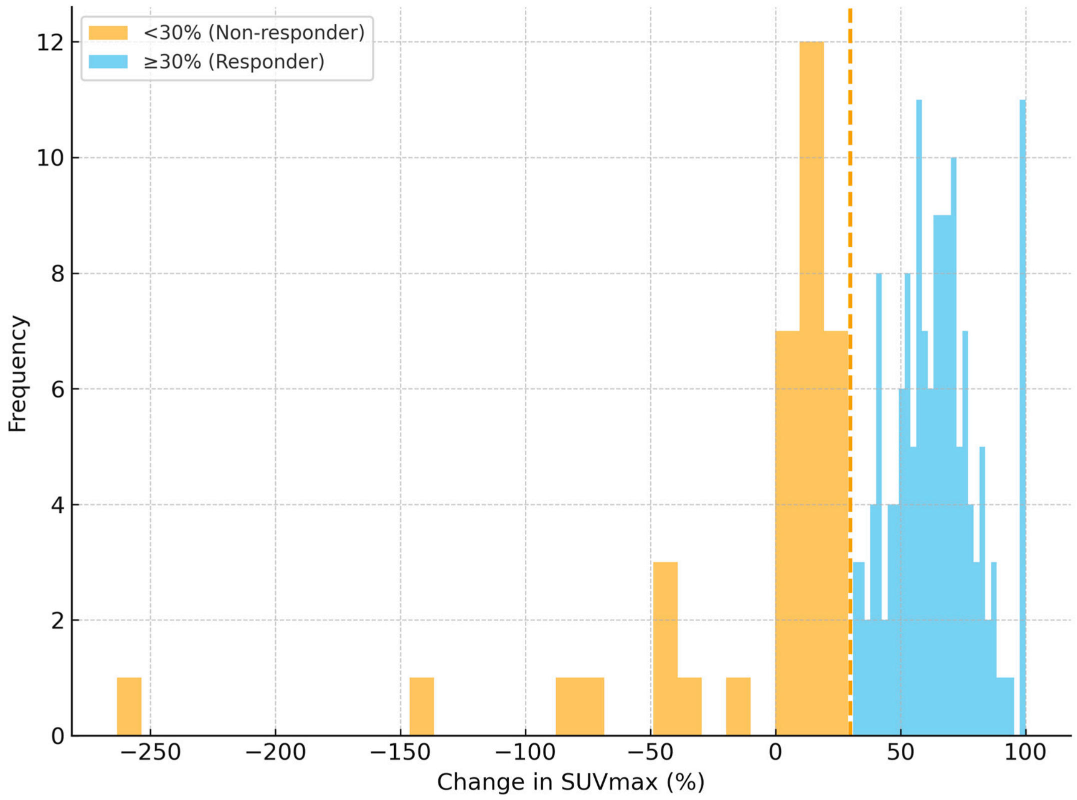

Overlaid histograms illustrating the distribution of percentage change in SUVmax among responders (≥30% reduction) and non-responders (<30% reduction). The dashed vertical line marks the 30% metabolic response threshold. This visualization highlights the distinct metabolic activity patterns between the two groups.

Supplementary Table S2. Metabolic response rates across metastatic subgroups

Metastatic Subgroup	Total (n)	Metastasis Present (n)	Responder% (Present)	No Metastasis (n)	Responder% (Absent)
**Bone metastasis**	203	147	**78.2%**	56	**67.9%**
**Visceral metastasis**	203	99	**69.7%**	104	**80.8%**
**Liver metastasis**	203	40	**57.5%**	163	**79.8%**
**Lung metastasis**	203	55	**78.2%**	148	**74.3%**
**Brain metastasis**	203	7	**28.6%**	196	**77.0%**

Metabolic response rates showed substantial heterogeneity across metastatic subgroups. Brain and liver metastases were associated with notably lower response rates, while bone-only and lung metastases demonstrated comparatively higher metabolic response proportions.

PET/CT Acquisition Protocol

PET imaging was performed using GE Discovery 710 PET/CT and GE Discovery IQ PET/CT systems (GE Healthcare, Milwaukee, WI, USA). Reconstruction was carried out using an ordered-subset expectation maximization (OSEM) algorithm with 3 iterations and 24 subsets. Images were reconstructed using a 256 × 256 matrix and a 5-mm Gaussian post-reconstruction filter. Identical reconstruction parameters were applied at both institutions. No formal EARL-based cross-calibration was performed due to the retrospective study design.

SUV measurements were normalized to body weight. SUVmax was derived from the most metabolically active lesion identified on baseline PET/CT and re-evaluated at interim imaging.

ROC Analysis

ΔSUVmax demonstrated modest discriminative ability for predicting progression, with an AUC of 0.65 (95% CI 0.57–0.73) (Figure 3). The optimal cut-off according to the Youden index was a 32.4% reduction, yielding a sensitivity of 35.5% and specificity of 91.1%. This threshold closely aligned with the predefined ≥30% response criterion used in the primary analyses.

Supplementary Figure S3. ROC curve for ΔSUVmax predicting progression

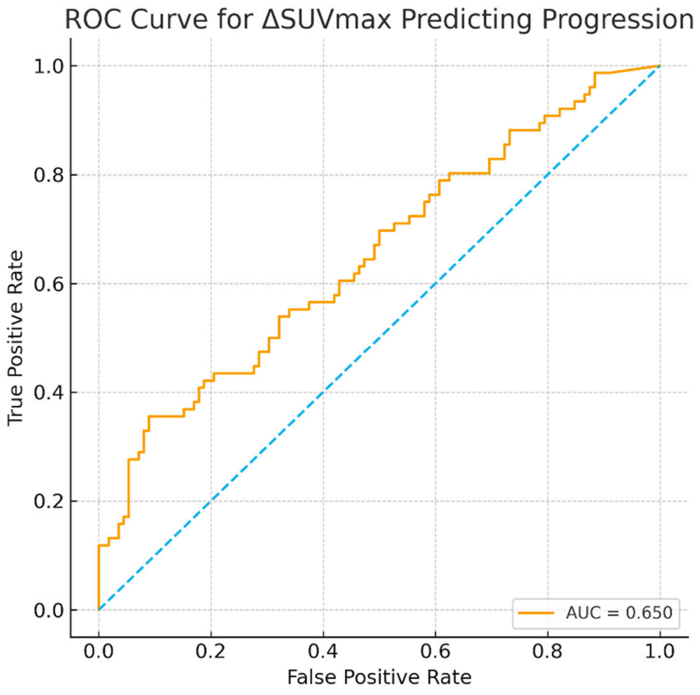

ΔSUV_max demonstrated modest discriminatory ability for predicting progression on a binary scale, with an AUC of 0.65 (95% CI 0.57–0.73). The optimal cut-off derived from the Youden index was a 32.4% reduction in SUV_max, yielding a sensitivity of 35.5% and a specificity of 91.1%. Importantly, this threshold closely aligned with the predefined ≥30% metabolic response criterion used in the primary analyses.

Although ROC-based sensitivity was limited—reflecting the multifactorial nature of endocrine resistance—the near-identical ROC-derived cut-off and the clear separation of survival curves indicate that ΔSUV_max carries substantially greater value as a time-to-event prognostic marker rather than as a standalone diagnostic classifier. This pattern is consistent with recent PET response studies evaluating early metabolic changes during CDK4/6 inhibitor therapy.

Supplementary Table S3. Center-stratified analysis of the association between metabolic response and progression-free survival

**Center**	**Responder (n)**	**Non-responder (n)**	**HR**	**p-value**
Center 1	92	35	0.47	<0.001
Center 2	61	15	0.33	<0.001

Hazard ratios are calculated comparing responders versus non-responders within each center.

Supplementary Figure S4. Kaplan–Meier curves for progression-free survival according to study center.

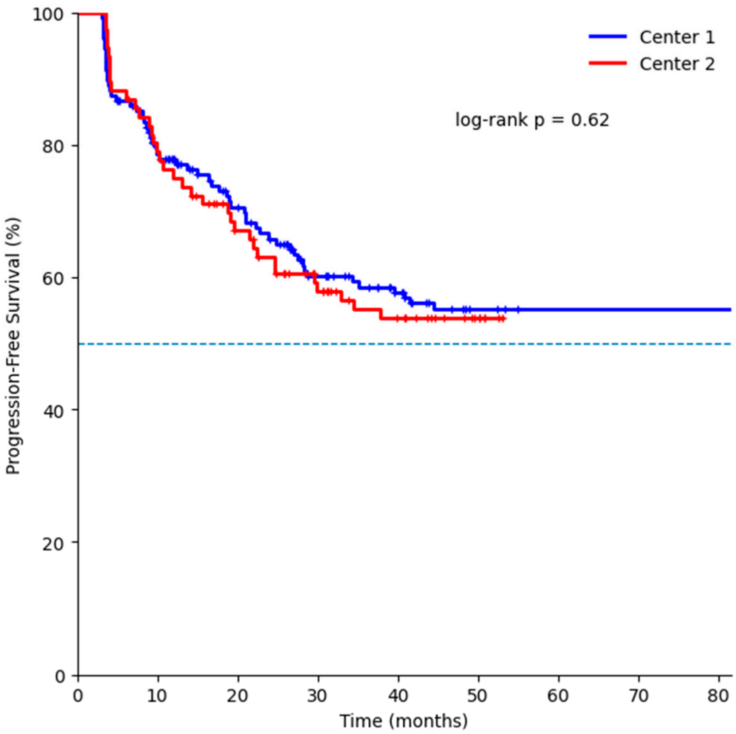

No significant difference in progression-free survival was observed between Center 1 and Center 2 (log-rank p = 0.62). Censoring is indicated by "+" marks.

Supplementary Table S4. Univariable Cox regression analysis of baseline and interim SUVmax in relation to progression-free survival (PFS) and overall survival (OS)

**Outcome**	**Predictor**	**(HR)**	**95% CI**	**p-value**
PFS	Baseline SUVmax (per 1 unit)	1.02	0.98–1.07	0.31
PFS	Interim SUVmax (per 1 unit)	1.20	1.14–1.26	<0.001
OS	Baseline SUVmax (per 1 unit)	1.01	0.95–1.08	0.73
OS	Interim SUVmax (per 1 unit)	1.07	1.01–1.13	0.02

Hazard ratios are derived from univariable Cox proportional hazards models. SUVmax was analyzed as a continuous variable.

Supplementary Figure S5. Kaplan–Meier analysis of progression-free survival according to metabolic response category.

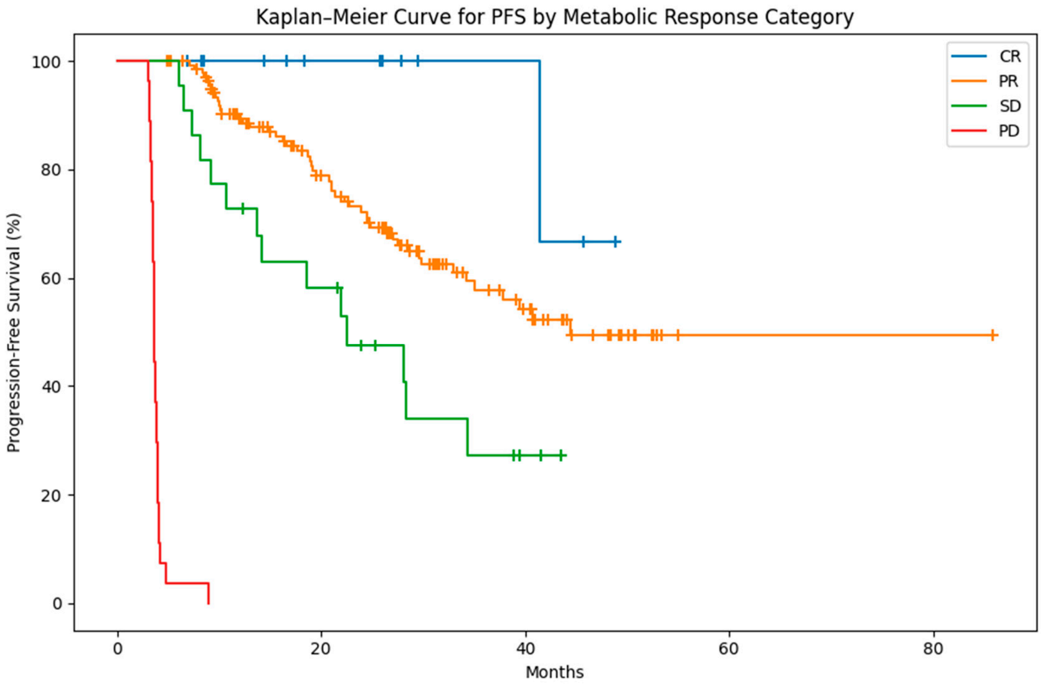

Kaplan–Meier curves demonstrate progression-free survival (PFS) stratified by interim metabolic response on ^18F-FDG PET/CT (CR, PR, SD, PD). Patients achieving complete or partial metabolic response had significantly prolonged PFS compared with those with stable or progressive disease (log-rank $p < 0.001$). Median PFS was not reached for CR and PR groups, whereas it was 22.5 months for SD and 3.7 months for PD. Tick marks indicate censored observations.

Supplementary Table S5. Distribution of metabolic response categories on interim PET/CT (n = 203)

Metabolic Response	n (%)	Median PFS (months)
Complete Response (CR)	13 (6.4%)	Not reached
Partial Response (PR)	141 (69.5%)	Not reached
Stable Disease (SD)	22 (10.8%)	22.5
Progressive Disease (PD)	27 (13.3%)	3.7

Overall comparison (log-rank test): $p < 0.001$

Footnote

Median PFS values were estimated using the Kaplan–Meier method. "Not reached" indicates that the survival curve did not fall below 50% during follow-up. P-values were calculated using the log-rank test across all four metabolic response categories
